# 

**Published:** 1855-09

**Authors:** 


VOL. VI. NEW SERIES. No. 9.

### THE NORTH-WESTERN MEDICAL AND SURGICAL JOURNAL:

EDITED BY

N. S. DAVIS, M.D.,

PROFESSOR OF PRINCIPLES AND PRACTICE OF MEDICINE AND CLINICAL MEDICINE IN RUSH MEDICAL COLLEGE; ONE OF THE PHYSICIANS TO THE MERCY HOSPITAL; FELLOW OF THE COLLEGE OF PHYSICIANS AND SURGEONS, NEW YORK; MEMBER OF THE MEDICAL SOCIETY OF THE STATE OF NEW YORK, AND OF THE STATE OF ILLINOIS; MEMBER OF THE AMERICAN MEDICAL ASSOCIATION, ETC. ETC.

AND

H. A. JOHNSON, A.M., M.D.

PROFESSOR OF MATERIA MEDICA AND MEDICAL JURISPRUDENCE IN RUSH MEDICAL COLLEGE, ONE OF THE SURGEONS OF MERCY HOSPITAL, MEMBER OF THE AMERICAN MEDICAL ASSOCIATION, MEM. OF ILL. STATE MED. SOCIETY, &C. &C.

PUBLISHED MONTHLY,

AT TWO DOLLARS PER ANNUM IN ADVANCE.

SEPTEMBER, 1855.

CHICAGO:

J. F. BALLANTYNE, BOOK AND JOB PRINTER,

NEW POST-OFFICE BUILDINGS, COR. OF CLARK AND RANDOLPH-STS.,

OPPOSITE THE SHERMAN HOUSE.

VOL. XII. WHOLE SERIES, NO. 9.

